# Impacts of Chronic Alkalinity Stress on Growth, Physiology, Histology, and Muscle Quality in Qihe Crucian Carp (*Carassius carassius*)

**DOI:** 10.3390/ani16101536

**Published:** 2026-05-17

**Authors:** Liangyan Wang, Siyu Chen, Songtao Xu, Yundong Li, Limin Wu, Xue Tian, Xiao Ma, Wenge Ma, Khor Waiho, Xi Shi, Xuejun Li

**Affiliations:** 1 College of Fisheries, Henan Normal University, Xinxiang 453007, China; wangliangyan@htu.edu.cn (L.W.); 18856625647@163.com (S.C.); wulimin@htu.edu.cn (L.W.); tianxue@htu.edu.cn (X.T.); maxiao@htu.edu.cn (X.M.); mawenge@htu.edu.cn (W.M.); 2 Water Conservancy and Aquatic Products, Qingdao 266700, China; xst15725279667@163.com; 3 South China Sea Fisheries Research Institute, Chinese Academy of Fishery Sciences, Guangzhou 510300, China; liyd2019@163.com; 4 Higher Institution Centre of Excellence (HICoE), Institute of Tropical Aquaculture and Fisheries, University Malaysia Terengganu, Kuala Nerus 21030, Terengganu, Malaysia; waiho@umt.edu.my; 5 Observation and Research Station on Water Ecosystem in Danjiangkou Reservoir of Henan Province, Nanyang 474450, China

**Keywords:** saline-alkaline water, aquaculture, candidate species, nutritional composition

## Abstract

Saline-alkaline water is increasingly used for aquaculture. Qihe crucian carp (*Carassius carassius*) is one of the major economic aquacultural species in China; however, the impact of saline-alkaline water on this fish remains unclear. In this study, a 60 d chronic alkalinity stress experiment was conducted to investigate the effects on the growth, physiology, and muscle quality of Qihe crucian carp. One hundred and eighty juvenile fish (31.03 ± 0.71 g) were divided into three treatments (three replicate tanks per treatment, 20 fish per tank): a control group (freshwater), a CA20 group (20 mmol/L), and a CA40 group (40 mmol/L). Despite no effect on survival, alkalinity stress significantly impaired growth performance in both the CA20 and CA40 groups, leading to reductions in final body weight, weight gain rate, and specific growth rate. Similarly, the feed efficiency decreased with increasing alkalinity. Compared to the control group, the alkalinity groups exhibited elevated serum Na^+^ levels and higher gill Na^+^/K^+^-ATPase activity, but reduced Ca^2+^/Mg^2+^-ATPase activity. For histology, chronic alkalinity stress induced several changes in the gills, including wider filaments, shortened lamellae, and the increase in interlamellar distance. Regarding muscle nutritional composition, the contents of crude protein and amino acids and the proportions of polyunsaturated fatty acids showed a downward trend with rising alkalinity. Additionally, alkalinity stress significantly decreased muscle fiber density and hardness. Taking into account both growth performance and muscle quality, the suggested aquacultural alkalinity should be controlled below 20 mmol/L for Qihe crucian carp. In conclusion, these findings supported Qihe crucian carp as a promising candidate species for saline-alkaline water aquaculture, providing a scientific basis for the utilization of this special water resource.

## Simple Summary

Due to the shortage of freshwater resources, saline-alkaline water is increasingly being used in aquaculture. Qihe crucian carp, an important economic aquaculture species in China with high alkalinity tolerance, is primarily farmed in northern Henan province, bringing high economic benefits to local famers. This study tested the effects of chronic alkalinity stress (20 and 40 mmol/L over 60 days) on this fish species. Although higher alkalinity reduced growth performance, feed efficiency, altered gill structure and ion regulation, and decreased meat quality (protein, amino acids, fatty acids, and muscle texture) to some extent, the survival rate was not affected, and the fish still maintained relatively high nutritional quality. To maintain good growth performance and meat quality, alkalinity should be controlled below 20 mmol/L. In conclusion, Qihe crucian carp is a suitable candidate for farming in moderately saline-alkaline water.

## 1. Introduction

Global freshwater resources are facing growing scarcity due to population expansion, climate change, and water pollution [1,2]. Saline-alkaline water is a distinctive non-marine saline resource formed through scarce rainfall, drought, or prolonged evaporation. This resource has a broad global distribution, spanning more than 100 countries and regions, and accounts for a notable portion of water resources worldwide [3,4]. China encompasses an estimated 45.87 million hectares of saline-alkaline water bodies, which are primarily located in its northeastern, northwestern, and coastal provinces [5,6]. However, because of its high pH and carbonate alkalinity, unbalanced major ion ratios, and poor buffering capacity, this water cannot be easily utilized for crop irrigation or as potable water [7]. Consequently, most of these water resources remain untapped [8]. Recently, researchers have attempted to establish aquaculture in saline-alkaline water to reduce freshwater consumption and promote the utilization of available resources [6,9]. Currently, certain low saline-alkaline waters have been successfully utilized through the transplantation and domestication of selected freshwater fish, shrimp, and crab species [3]. However, moderate-high saline-alkaline waters remain largely untapped [10]. Therefore, it is crucial to screen suitable aquatic species for culture in medium-high saline-alkaline water environments.

As a primary stressor in saline-alkaline water, carbonate alkalinity adversely affects key biological processes in aquatic animals, from growth and physiological metabolism to tissue function and nutritional value [11]. For example, alkaline stress reduced the activities of intestinal proteases, lipases, and amylases, and significantly impaired the growth performance of grass carp (*Ctenopharyngodon idella*) [12]. Furthermore, under alkaline stress, *Scatophagus argus* exhibited excessive production of reactive oxygen species (ROS), which induced oxidative stress, disrupted the balance within the antioxidant system, and subsequently caused structural and functional damage to gill tissues [13]. Conversely, research has demonstrated that moderate levels of carbonate alkalinity could help maintain the stability of water pH and ions, reduce the toxicity of metal ions in the water, as well as promote fish growth [14]. However, in aquatic animals, excessive alkalinity can disrupt acid–base balance, impair antioxidant and ammonia metabolism functions, trigger metabolic alkalosis, and ultimately result in growth suppression [15,16]. Generally, the acclimation of fish to alkaline stress is primarily mediated by physiological modifications in gill morphological characteristics and osmoregulatory functional capacities [17,18]. For example, *Paramisgurnus dabryanus* and *Lateolabrax maculatus* responded to alkalinity stress with alterations in gill lamellar length and interlamellar distance [7,19]. And alkalinity stress caused significant physiological stress in red tilapia (*Oreochromis spp*.), leading to alterations in gill structure and affecting antioxidant defense, metabolism, and ion balance [20]. Furthermore, the gills can adapt to alkalinity stress by altering the number and distribution of epithelial cells (EPCs), pillar cells (PCs), and mitochondria-rich cells (MRCs) [21]. Abundant in the gills, Na^+^/K^+^-ATPase (NKA) and Ca^2+^/Mg^2+^-ATPase (CMA) are key players in ion transport and gradient maintenance. For example, in *Piaractus mesopotamicus*, gill NKA activity was significantly increased when exposed to alkaline waters [22]. Thus, the activities of these enzymes typically changed when aquatic animals were subjected to alkalinity stress [6].

Saline-alkaline water environments can disrupt key physiological processes in aquatic animals, such as osmotic homeostasis, energy metabolism, and gut microbiota [10]. These disruptions can alter the synthesis and decomposition of muscle protein and fat, ultimately affecting nutritional quality [12,14]. However, an appropriate saline-alkaline water environment can improve the muscle nutritional composition of aquatic animals, thereby enhancing their overall nutritional quality. Specifically, following chronic exposure to 14 mmol/L alkalinity stress, the muscle of largemouth bass (*Micropterus salmoides*) exhibited significant increases in the concentrations of most amino acids, as well as in the contents of total amino acid (TAA), essential amino acid (EAA), and delicious amino acid (DAA). The composition of DAAs, including glycine, alanine, glutamic acid, and aspartic acid, is the main factor determining the flavor of fish meat. This indicates an improvement in amino acid nutritional quality [23,24]. Similarly, a study revealed that red drum (*Sciaenops ocellatus*) reared in low saline-alkaline water exhibited significantly higher levels of crude fat, ash, protein, umami and sweet amino acids, and free amino acids, concurrently with a lower proportion of bitter amino acids in muscle [25]. Studies on Nile tilapia (*Oreochromis niloticus*) also confirmed that low alkalinity water can enhance muscle nutritional value and flavor [26]. Nevertheless, research on the effects of medium-high alkalinity levels on fish muscle quality remains limited.

The crucian carp (*Carassius carassius*), a major economic species in Chinese freshwater aquaculture with an annual output of 2.82 million tons in 2024, ranking fifth after grass carp (*Ctenopharyngodon idella*, 6.16 million tons), silver carp (*Hypophthalmichthys molitrix*, 3.91 million tons), bighead carp (*H. nobilis*, 3.49 million tons), and common carp (*Cyprinus carpio*, 2.94 million tons), is also a classic model in toxicology due to its high sensitivity to environmental pollutants [27]. A previous study indicated that alkalinity stress altered serum immune parameters, suppressed antioxidant enzyme activities, and caused oxidative damage in crucian carp [17]. Furthermore, long-term high-alkalinity stress can disrupt hepatic antioxidant capacity and lipid metabolism, while triggering apoptosis and immune responses [28,29]. This stress can also induce severe kidney injury by disrupting kidney antioxidant defenses and energy homeostasis, and suppressing protein catabolism and metabolism [30]. Although the physiological, biochemical, and molecular responses of crucian carp to alkaline stress have been extensively characterized, it is still unclear whether long-term alkaline stress affects their important economic traits such as growth performance and muscle quality.

Qihe crucian carp is a local subspecies of *C. gibelio* that evolved through a long-term ecological adaptation and is characterized by its broad back and delicious meat [31]. It is widely farmed in the Central Plains region of China. In recent years, some farmers have begun to develop aquaculture of Qihe crucian carp in low saline-alkaline waters and have achieved excellent economic benefits. However, it remains unknown whether the growth, morphology, physiology, histology, and muscle quality of Qihe crucian carp would change under medium-high saline-alkaline water conditions. Therefore, a 60 d chronic alkalinity stress experiment was conducted on juvenile Qihe crucian carp to assess whether it was suitable as an ideal aquaculture species for medium-high saline-alkaline waters and to provide a theoretical basis for the proper utilization of these resources.

## 2. Materials and Methods

### 2.1. Experimental Fish

All experimental juvenile Qihe crucian carp were obtained from the Aquaculture Base of Henan Normal University (Xinxiang, China). All these fish were from the same batch of fry reproduced during the same year. Prior to the experiment, the juvenile fish were temporarily held in cement tanks for 14 d acclimation. During the acclimation period, the dissolved oxygen was maintained at ≥7.0 mg/L, ammonia nitrogen at <0.1 mg/L, pH at 7.7–8.1, temperature at 22–26 °C, and no additional alkalinity or salinity was added during acclimation. Throughout the acclimation, the juvenile fish were fed to satiation with a commercial diet twice daily. Afterwards, one hundred and eighty healthy juveniles of uniform body weight (31.03 ± 0.71 g) were randomly chosen for the chronic alkalinity stress experiment.

### 2.2. Experimental Design and Daily Management

The alkalinity stress experiment was conducted in an indoor, temperature-controlled greenhouse using a static aquaculture system with 200 L tanks. According to our previous study, the safe concentration of the alkalinity stress for juvenile Qihe crucian carp was 20.79 mmol/L [32]. Therefore, a total of 180 fish, after initial body weight (IBW) measurement, were randomly distributed into three groups with three replicates each (20 fish per replicate): a freshwater control (2.56 mmol/L, equivalent to 215 mg/L), a CA20 group (20 mmol/L, equivalent to 1680 mg/L), and a CA40 group (40 mmol/L, equivalent to 3340 mg/L). Fish in the control group were reared in aerated tap water, while the alkalinity treatments were prepared by adding NaHCO_3_ to freshwater.

Throughout the 60 d experiment, the fish were fed manually until they appeared to be satiated twice daily (at 09:00 and 16:00) with a commercial expanded diet purchased from Henan Tongwei Feed Co., Ltd. (Xinxiang, China). The diet was formulated to contain crude protein ≥ 35.0%, crude lipid ≥ 3.0%, crude fiber ≤ 7.0%, crude ash ≤ 16.0%, lysine ≥ 1.8%, moisture ≤ 12.0%, and total phosphorus ≥ 1.1%. Feed intake (FI) was recorded daily. Mortality was checked each morning before feeding. To maintain water quality, 50% of the tank water was replaced two hours after morning feeding and continuous aeration was provided throughout the experiment. Key water parameters were monitored daily to keep them consistent with the temporary rearing conditions.

### 2.3. Assessment of Growth, Feed Efficiency, and Morphological Characteristics

After the experiment, all fish in each tank were starved for 24 h, and then counted and weighed to calculate final body weight (FBW), survival rate (SR), specific growth rate (SGR), feeding rate (FR), weight gain rate (WGR), and feed conversion ratio (FCR). Ten fish per tank were then anesthetized with MS-222 (100 mg/L). Their body weight (BW) and thirteen morphological parameters were measured using an electronic balance (0.01 g) and a vernier caliper (0.01 mm) following the methods described by Shi et al. (2022) [31]. The measured morphological traits were: full length (FL), head length (HL), head length behind eyes (HLBE), distance between eyes (DBE), body thickness (BT), body length (BL), snout length (SL), body height (BH), caudal length (CL), caudal peduncle length (CPL), caudal fin length (CFL), caudal peduncle height (CPH), and trunk length (TL). Subsequently, twelve of these morphological traits (excluding BL) were standardized against BL. Thereafter, three additional fish per tank were anesthetized. Their BW and BL were recorded before they were dissected on ice to collect and weigh the visceral mass and hepatopancreas, allowing for the calculation of the condition factor (CF), viscerosomatic index (VSI), and hepatosomatic index (HSI). Finally, non-muscular parts (skin, viscera, gills, scales, bones) were removed to determine the meat content (MC). The calculation formulas for all aforementioned indices were provided below:

SR = (final number of fish/initial number of fish) × 100%

WGR = (FBW − IBW)/FBW × 100%

SGR = (Ln FBW − Ln IBW)/days × 100%

FCR = FI/(FBW − IBW)

FR = FI/[(FBW + IBW)/2]/days × 100%

HSI = liver weight/BW × 100%

VSI = viscera weight/BW × 100%

CF = BW/BL^3^ × 100%

MC = (meat weight/BW) × 100%

### 2.4. Sample Collection

For blood and gill tissue collection, three fish were randomly sampled from each tank. After anesthesia with MS-222, blood was drawn from the caudal vein and stored at 4 °C for 24 h. The blood sample was then centrifuged (5000 rpm, 10 min, 4 °C) to obtain serum for ion analysis. Gill tissues were collected and divided into two portions: one was flash-frozen for subsequent NKA and CMA activity assays, and the other was fixed in 4% paraformaldehyde (PFA) for histological examination. Additionally, nine fish per tank were anesthetized and dissected. Muscle tissues were then collected and divided into three parts: one part was fixed in 4% PFA for histological observation and analysis; the second portion was reserved for texture profile analysis and water-holding capacity assessment, and the third part was stored at −80 °C for subsequent analysis of proximate composition, amino acid contents, and fatty acid contents. To ensure the reliability and consistency of the experimental results, the sampling site for each specific analysis was kept as consistent as possible across all individuals.

### 2.5. Analysis of Serum Ion Concentration and ATPase Activities

Serum concentrations of Na^+^, K^+^, Ca^2+^, Mg^2+^, and Cl^−^ were determined using an automatic biochemical analyzer (Rayto, Shenzhen, China) at wavelengths of 405, 630, 660, 540, and 510 nm, respectively, with three replicates per sample. For gill tissues, thawed samples were homogenized in 0.85% normal saline and centrifuged (6000 rpm, 10 min, 4 °C). The resulting supernatant was used to determine total protein concentration, NKA activity, and CMA activity with commercial kits. These kits were obtained from Nanjing Jiancheng Bioengineering Institute (Nanjing, China), with product numbers of A045-4-2, A070-2-2, and A070-3-2, respectively.

### 2.6. Histological Analysis

Histological analysis was performed using hematoxylin and eosin (H&E) staining, and the detailed procedures were provided below. Gill and muscle samples from three fish randomly selected in each group were sectioned, and two slices were prepared from each tissue. The tissues preserved in 4% PFA were rinsed with phosphate-buffered saline (PBS). They were then dehydrated in a graded ethanol series, cleared in xylene, embedded in paraffin, and sectioned at 5 μm thickness. The sections were stained with H&E, followed by dehydration, clearing, and mounting with neutral resin. Stained sections were imaged with a digital Zeiss microscope (Zeiss, Oberkochen, Germany). Morphometric analysis was performed with Image J software (version 1.52a) to measure gill lamellar length, thickness, and interlamellar distance, as well as muscle fiber diameter and density. For each parameter, measurements were repeated three times per section to ensure accuracy.

### 2.7. Analysis of Muscle Nutritional Composition, Amino Acid and Fatty Acid Content

The proximate composition, amino acid contents, and fatty acid contents of muscle samples were analyzed following the method of Jia et al. (2022) [33]. Specifically, moisture content was determined by freeze-drying, and crude ash was measured by burning in a muffle furnace at 550 °C for 5 h. Crude protein was analyzed using a Kjeldahl nitrogen analyzer, and crude fat was extracted using a Soxhlet extractor. Amino acid profiling was performed on an A300-advanced automatic amino acid analyzer (Germany). Fatty acid composition and content were determined by gas chromatography (Trace 1310-ISQ, Thermo, Waltham, MA, USA) using the area normalized method.

### 2.8. Determination of Muscle Physical Properties

Muscle water-holding capacity, texture profile, and pH were measured following the method described by Jia et al. (2022) [33]. All measurements were performed in triplicate per group, with three fish sampled per replicate. For texture analysis, muscle samples were cut into 1.0 cm^3^ cubes, boiled for 5 min, and then evaluated using a TA-XT plus texture analyzer (Stable Micro System Ltd., Surrey, UK). Muscle pH was measured with a PB-10 pH meter after homogenizing 1 g of muscle in 9 mL of deionized water.

### 2.9. Statistical Analysis

All data were analyzed by using SPSS software (version 23.0) and presented as mean ± standard deviation (SD). Normality was assessed using the Shapiro–Wilk test. Differences among groups were evaluated by one-way analysis of variance (ANOVA), followed by Duncan’s post hoc test for multiple comparisons. A significance level of *p* < 0.05 was considered statistically significant.

## 3. Results

### 3.1. Growth and Feed Efficiency

**Figure 1:**
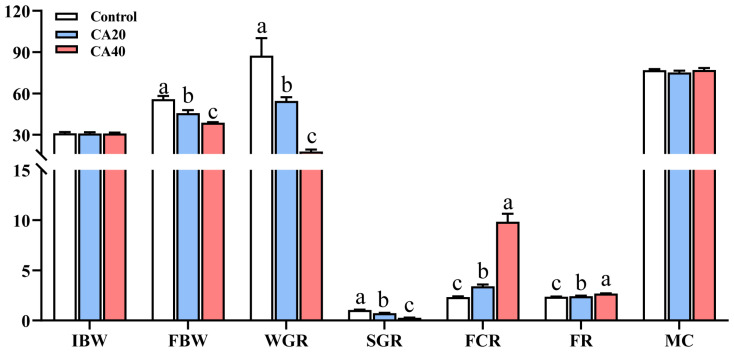
Growth performance and feed efficiency of Qihe crucian carp *Carassius carassius* after 60 d alkalinity stress. IBW (g): initial mean body weight; FBW (g): final mean body weight; WGR (%): weight gain rate; SGR (%): specific growth rate; FCR: feed conversion ratio; FR (%): feeding rate; MC (%): meat content. Data are presented as mean ± SD, and the bars marked with different letters indicate significant difference (*p* < 0.05).

All experimental groups exhibited 100% survival. Compared with the control group, FBW, WGR, and SGR were significantly reduced, and FCR and FR were significantly increased in the alkalinity-treated groups (*p* < 0.05). Additionally, the FBW, WGR, and SGR were significantly lower in the CA40 group than those in the CA20 group (*p* < 0.05). No significant difference in MC was observed among the groups (Figure 1; *p* > 0.05).

### 3.2. Morphological Characteristics

**Figure 2:**
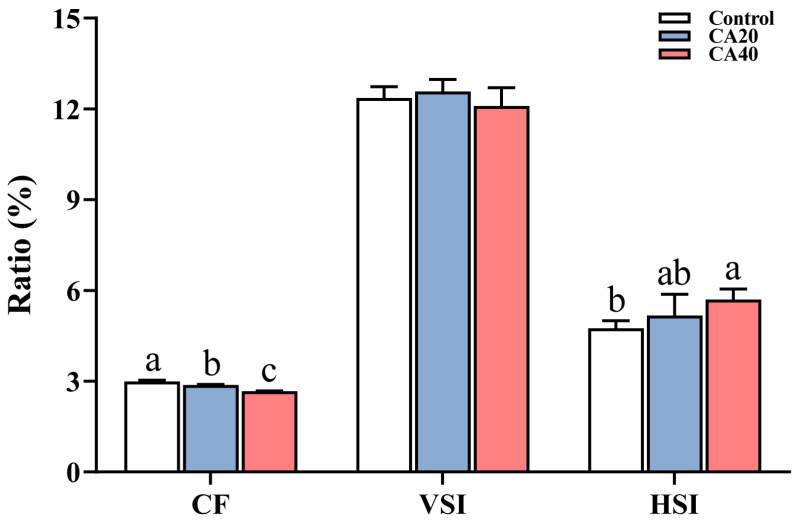
Morphological parameters of Qihe crucian carp *Carassius Carassius* after 60 d alkalinity stress. CF (%): condition factor; VSI (%): viscerosomatic index; HSI (%): hepatosomatic index. Data are presented as mean ± SD, and the bars marked with different letters indicate significant difference (*p* < 0.05).

The results revealed an inverse relationship between alkalinity concentration and CF, but a positive relationship with HSI (Figure 2). No significant differences were observed in VSI among the groups (*p* > 0.05). Regarding morphology parameters (Table 1), the CA20 group exhibited significant reductions only in SL and CPH compared to the control group (*p* < 0.05). In contrast, the CA40 group displayed a broader range of alterations, with significant increases in FL and HLBE, alongside significant decreases in BH, BT, SL, DBE and CPH (*p* < 0.05).

### 3.3. Serum Ion Concentrations

**Figure 3:**
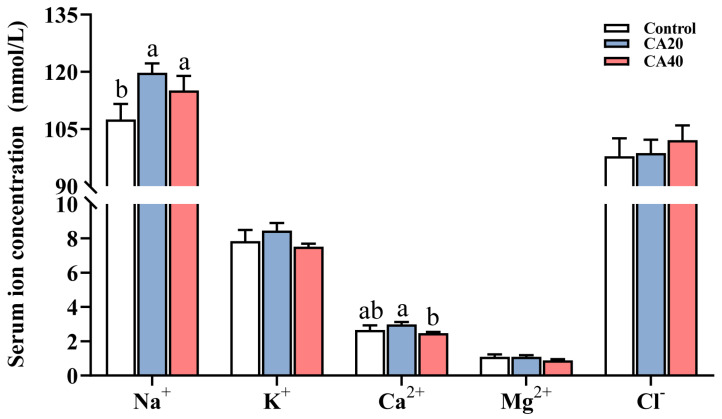
Serum ion concentrations of Qihe crucian carp *Carassius Carassius* after 60 d alkalinity stress. Data are presented as mean ± SD, and the bars marked with different letters indicate significant difference (*p* < 0.05).

Chronic alkalinity stress did not significantly alter serum K^+^, Mg^2+^, Cl^−^ concentrations compared to the control group (Figure 3; *p* > 0.05). Alkalinity stress significantly improved the concentration of Na^+^ (*p* < 0.05). Additionally, the concentration of Ca^2+^ in the CA20 group was significantly higher than that in the CA40 group (*p* < 0.05).

### 3.4. Gill NKA and CMA Activities

**Figure 4:**
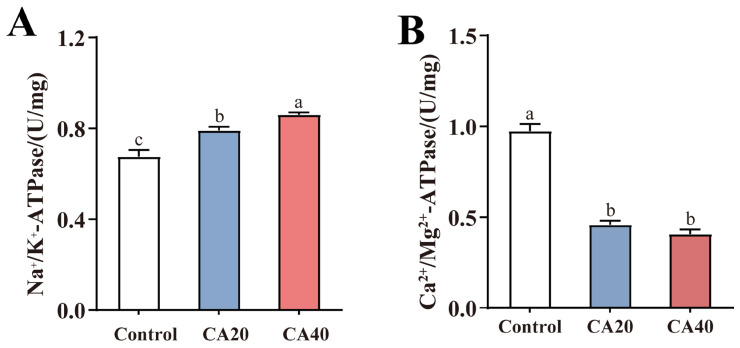
NKA and CMA activities in gills of Qihe crucian carp *Carassius Carassius* after 60 d alkalinity stress. (**A**) NKA: Na^+^/K^+^-ATPase; (**B**) CMA: Ca^2+^/Mg^2+^-ATPase. Data are presented as mean ± SD, and the bars marked with different letters indicate significant difference (*p* < 0.05).

After 60 d of alkalinity stress, gill NKA and CMA activities exhibited opposing trends (Figure 4). NKA activity showed a significant increasing trend with rising alkalinity levels (Figure 4A; *p* < 0.05). In contrast, CMA activity was significantly reduced in both alkalinity-treated groups compared to the control (Figure 4B; *p* < 0.05).

### 3.5. Gill Histology and Structural Features

**Figure 5:**
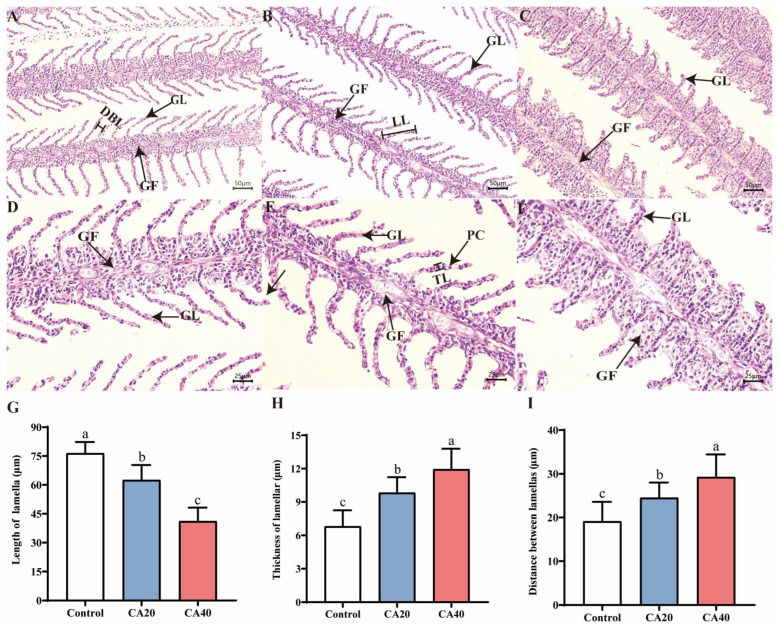
Histological structure and characteristics of gills of Qihe crucian carp *Carassius carassius* after 60 d alkalinity stress. (**A**–**C**) Control, CA20, and CA40 groups at 200× magnification, respectively. (**D**–**F**) Control, CA20, and CA40 groups at 400× magnification, respectively; (**G**–**I**) quantitative measurements of lamellar length, thickness and interlamellar distance, respectively. GL: gill lamella; GF: gill filament; BC: blood cell; PC: pillar cell; PVC: pavement cell. MRC: mitochondria-rich cell. Data are presented as mean ± SD, and the bars marked with different letters indicate significant difference (*p* < 0.05).

After 60 d of culture, the gills of the control group displayed a typical leaf-like branching structure, with well-arranged and evenly spaced lamellae (Figure 5A,D). In contrast, alkalinity stress induced distinct structural alterations (Figure 5B,C,E,F), including shortened and thickened lamellae (Figure 5G,H) and widened interlamellar spacing (Figure 5I). Furthermore, with increasing alkalinity, the CA20 group exhibited vasodilation and rupture of gill filament vascular cells (Figure 5B,E). The CA40 group showed similar symptoms, accompanied by EPC detachment, an increased number of MRCs, and swelling at the distal ends of the gill lamellae (Figure 5C,F).

### 3.6. Nutritional Composition, Amino Acid Content, and Fatty Acid Content of Muscle

The CA20 group exhibited significantly lower moisture content than the control and CA40 groups, while the CA 40 group showed a significantly higher crude ash content than the control and CA20 groups (Table 2; *p* < 0.05). Furthermore, the CA40 group displayed significant reductions in both crude protein and crude fat contents (*p* < 0.05).

**Figure 6:**
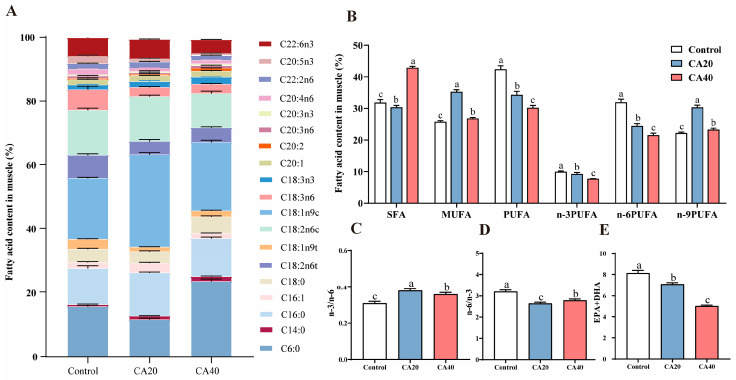
Fatty acid composition and content in Qihe crucian carp *Carassius Carassius* muscle after 60 d alkalinity stress. (**A**) Muscle fatty acid content; (**B**) summary of different types of fatty acids; (**C**) ratio of n-3 and n-6; (**D**) ratio of n-6 and n-3; (**E**) content of EPA + DHA. Data are presented as mean ± SD, and the bars marked with different letters indicate significant difference (*p* < 0.05).

Regarding amino acid profiles (Table 3), most amino acids, including Asp, Glu, Arg, Met, Leu, Phe, and Tyr, were significantly reduced in the alkalinity-treated groups compared to the control group (*p* < 0.05). A notable decline in TAA, EAA, and DAA was also observed with increasing alkalinity (*p* < 0.05). Furthermore, while the EAA/TAA and EAA/NEAA ratios in the CA20 group were significantly lower than those in the CA40 group (*p* < 0.05), and they did not differ significantly from the control group (*p* > 0.05). A total of 19 fatty acids were detected, comprising four saturated fatty acid (SFA), four monounsaturated fatty acids (MUFA), and eleven polyunsaturated fatty acids (PUFA) (Figure 6A). With increasing alkalinity, the levels of PUFA, n-6 PUFA, n-3 PUFA, eicosapentaenoic acid (EPA, C20:5n-3), and the sum of EPA and docosahexaenoic acid (DHA, C22:6n-3) were significantly reduced (Figure 6; *p* < 0.05). In contrast, the MUFA content was markedly elevated in both alkalinity-treated groups compared to the control group (Figure 6B; *p* < 0.05).

### 3.7. Muscle Water-Holding Capacity and Textural Properties

**Figure 7:**
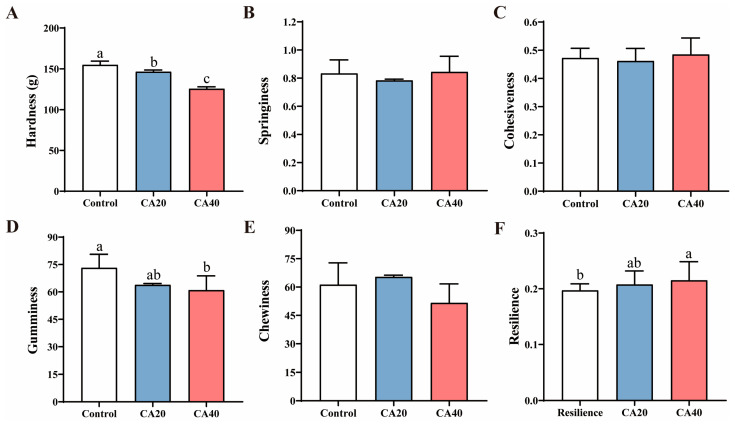
Textural characteristics of Qihe crucian carp *Carassius carassius* muscle after 60 d alkalinity stress. (**A**) Hardness; (**B**) springiness; (**C**) cohesiveness; (**D**) gumminess; (**E**) chewiness; (**F**) residence. Data are presented as mean ± SD, and the bars marked with different letters indicate significant difference (*p* < 0.05).

Compared to the control group, both alkalinity-treated groups exhibited a significant decline in muscle water-holding capacity, as evidenced by reduced pH and stored loss, alongside increased cooked rate and frozen leakage (Table 4; *p* < 0.05). This trend was further pronounced in the CA40 group, which showed the highest drip loss and centrifugal loss. Although liquid loss did not differ significantly among groups (*p* > 0.05), muscle texture softened with increasing alkalinity, characterized by decreased hardness and gumminess but increased resilience (Figure 7).

### 3.8. Histological Characteristics of Muscle

**Figure 8:**
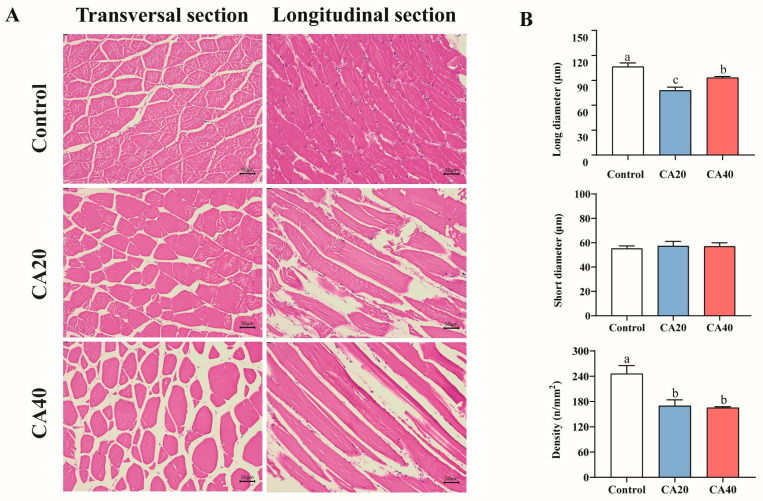
Histological characteristics of muscle tissue in Qihe crucian carp *Carassius carassius* after 60 d alkalinity stress. (**A**) Transversal and longitudinal sections of dorsal muscles; (**B**) muscle fiber dimensions (long and short diameter) and density. Data are presented as mean ± SD, and the bars marked with different letters indicate significant difference (*p* < 0.05).

Compared to the control group, both the CA20 and CA40 groups exhibited marked alterations in muscle tissue structure and fiber density (Figure 8). In the control group, muscle fibers were tightly packed and well-organized (Figure 8A), whereas increasing alkalinity led to enlarged inter-fiber spacing, irregular fiber arrangement, and a looser structure (Figure 8A). Both alkalinity-treated groups showed significantly reduced muscle fiber long diameter and density (Figure 8B; *p* < 0.05).

## 4. Discussion

Alkalinity represents a major environmental stressor for freshwater fish, as it can induce respiratory and metabolic alkalosis, severely compromising key physiological processes such as growth, survival, and reproduction [28,34]. The survival capability of aquatic animals in saline-alkaline water is the most critical indicator for evaluating their potential as candidate species for saline-alkaline water aquaculture [23]. And the ability of alkalinity tolerance varied among different species. For instance, juvenile silver crucian carp exposed to acute alkalinity stress at 30 mmol/L all died within 48 h, while 30 mmol/L alkalinity significantly reduced growth performance and feed efficiency in grass carp (*C. idella*) juveniles and altered gill lamellar structure [12,35]. In contrast, largemouth bass exposed to an alkalinity environment of 28 mmol/L did not affect their survival [23]. These results indicated that the ability of alkalinity tolerance varied among different species. In the present study, Qihe crucian carp exhibited 100% SR after 60 d of exposure to 20 and 40 mmol/L alkalinities, indicating a strong tolerance to alkalinity and suggesting that their alkalinity tolerance mechanisms and processes deserve a more precise description.

Previous studies have demonstrated that several economic aquatic animals, such as Nile tilapia and largemouth bass, can survive and grow normally in low-alkalinity waters (<10 mmol/L) which were suitable for aquaculture [14,23]. However, most medium-high alkalinity waters (>10 mmol/L) were still in a barren and idle state, which needed to be developed [23,36]. In aquaculture, growth performance served as a direct indicator of both the output and economic benefits. Research has shown that carbonate alkaline stress can significantly inhibit the growth of fish. For example, the growth performance of Songpu mirror carp was significantly inhibited in an alkaline environment [5,37]. Specifically, growth may be enhanced within an optimal range of low alkalinity but be inhibited once alkalinity exceeds a certain threshold [12,15]. Our results demonstrated that increasing alkalinity significantly impaired the growth performance of Qihe crucian carp, as evidenced by reduced FBW, WGR, and SGR. This pattern mirrors trends reported in grass carp and largemouth bass [12,23]. Additionally, the CA40 group exhibited poorer growth performance compared to the CA20 group, indicating the existence of the dose effect of alkalinity stress, as observed in largemouth bass [23]. Based on previous studies, the observed growth suppression under high-alkalinity conditions can be attributed to several potential mechanisms. Firstly, increased alkalinity may elevate the energy expenditure required for osmoregulation and acid–base balance, thereby reducing the energy available for growth metabolism and ultimately impairing growth performance [35,38]. Additionally, structural damage to the intestine and liver from alkalinity stress may adversely affect their physiological function, leading to reduced nutrient absorption [39]. Furthermore, it was speculated that alterations in the gut microbiota composition may be one of the potential factors contributing to the reduced feed efficiency observed in this study [14,40]. However, the molecular and physiological mechanisms by which alkalinity stress impairs the growth of Qihe crucian carp remain largely unknown and warrant further investigation in the future.

Research has shown that environmental stress induced energy reallocation in fish, wherein the metabolic energy allocated to coping with environmental pressure increased, consequently reducing the energy available for growth [41]. Under alkaline conditions, fish need to activate ATPase activities involved in ion regulation and acid–base balance, thereby incurring energetic costs and diminishing the energy available for growth [6]. Our observations of increased NKA activity and HSI, coupled with the decrease in CF in the CA20 and CA 40 groups, supported this energy reallocation hypothesis. Morphometric indices are effective tools for assessing growth status, health, and aquaculture outcomes [35,42]. This study examined changes in the external morphology in Qihe crucian carp under alkalinity stress. The results further revealed that the CA40 group exhibited decreased BH and BT, suggesting that the higher alkalinity induced a leaner body shape, which corroborated the CF results. This phenomenon may stem from the catabolism of proteins and fats under alkaline stress to meet energy demands [14]. To date, research on alkalinity-induced morphological alterations has been limited, primarily focusing on largemouth bass [23]. Our observations on the external morphological responses of Qihe crucian carp represent a valuable addition to the scientific literature.

The gill, as the primary organ for osmoregulation, ion transport, respiration, ammonia nitrogen excretion, and gas exchange, undergoes comprehensive structural and functional remodeling to acclimate to high-alkalinity environments, encompassing both macroscopic and microscopic adjustments [10,43]. Macroscopically, fish primarily adapted to alkalinity stress through changes in structures and quantities such as gill rakers, lamellae, and filaments. For instance, *Leuciscus waleckii* from Dali Lake developed wider filaments, elongated lamellae, and enlarged interlamellar spaces under 30–50 mmol/L alkalinity, while *P. dabryanus* and *L. maculatus* exhibited increased lamellar height and spacing [7,10,19]. In the present study, Qihe crucian carp displayed shortened lamellae, thickened filaments, and widened interlamellar spacing. In addition, our study found that the alkalinity-treated groups exhibited vasodilation in the gill lamellae. Similarly, exposure of Nile tilapia to waterborne copper and cadmium has also been shown to induce vasodilation [44,45]. And such changes in vascular morphology may promote gas exchange and keep ion balance in fish [43]. In conclusion, Qihe crucian carp can adapt to the alkalinity environment through morphological alterations in gill tissue.

At the microscopic level, the gill tissue of fish is mainly composed of various cell types such as MRCs, mucous cells (MCs), pavement cells (PVCs), PCs, and blood cells (BCs). In alkaline water, HCO_3_^−^ and CO_3_^2−^ directly act on the gill epidermal cells (MRCs, PVCs, MCs, etc.), affecting their gas and ion exchange functions, interfering with ammonia nitrogen metabolism and acid–base balance, and causing ammonia poisoning and alkalosis in the fish body [46]. Studies on *L. waleckii* reported lamellar damage, epithelial detachment, and reduced MCs/PVCs under high alkalinity [47]. In the present study, we observed epithelial shedding and MRCs proliferation in Qihe crucian carp. These structural modifications in tissue organization and cell population may enhance gas diffusion efficiency in Qihe crucian carp under carbonate alkalinity stress, thereby improving oxygen uptake to counteract the adverse effects of high carbonate alkalinity on it [19]. Alterations in gill structure often led to changes in physiological function. In the present study, NKA activity in gills of Qihe crucian carp increased significantly with rising alkalinity, consistent with findings in Hefang crucian carp, largemouth bass and *Phoxinus lagowskii* [6,23,48]. Furthermore, the analysis of serum ions in this study revealed that, compared to the control group, the concentrations of K^+^, Mg^2+^, Cl^−^, and Ca^2+^ in the alkalinity-treated groups remained relatively stable, whereas the concentration of Na^+^ increased significantly. This suggested that in response to chronic alkalinity stress, Qihe crucian carp may maintain a higher Na^+^ concentration to regulate osmotic balance by upregulating NKA activity [10]. Conversely, CMA activity in gills of Qihe crucian carp significantly declined under high-alkalinity conditions, a pattern as also observed in juvenile silver crucian carp [35]. CMA is primarily responsible for actively pumping Ca^2+^ out of cells to maintain low intracellular Ca^2+^ concentrations [23]. However, serum Ca^2+^ concentrations showed no significant differences between the alkalinity-treated groups and the control group, suggesting that the fish may employ alternative calcium regulatory mechanisms to maintain blood calcium homeostasis [49].

Alkalinity stress not only impacts growth but also modifies muscle quality, a key determinant of aquaculture product value, which is influenced by both intrinsic and environmental factors [23,50]. Fish muscle quality is evaluated based on multiple indicators including nutritional composition, texture characteristics, color and flavor [51]. These attributes are influenced by both intrinsic factors (species, age, sex, etc.) and environmental conditions (temperature, pH, oxygen, salinity, alkalinity, etc.) [23,50,52]. The contents of moisture, crude protein, crude fat, and crude ash constitute key proximate components in muscle quality assessment. The present study found that, compared with the control group, the CA40 group showed significantly lower contents of crude fat and crude protein, whereas no significant difference was observed with the CA20 group. This finding indicated the existence of the dose effect of alkalinity stress in Qihe crucian carp. For Qihe crucian carp, 40 mmol/L alkalinity may represent a severe environmental challenge, causing it to expend a large amount of energy to regulate osmotic pressure, and during this process, the fat and protein in the muscle may be consumed for energy supply [28,38].

The profiles and levels of amino acids are closely linked to the nutritional quality of muscle protein. In response to environmental stress, free amino acids contribute to osmotic regulation, thereby directly impacting the amino acid profile in muscle tissue [26]. Our findings revealed that the content of TAA significantly decreased with the increase in alkalinity. This finding differed from the increased TAA content observed in red drum and largemouth bass under low saline-alkaline water [23,25]. When facing environmental alkalinity stress, Qihe crucian carp may consume amino acids as a direct energy source to maintain physiological functions, thereby declining amino acid levels in tissues [53]. Furthermore, the alkalinity stress led to a significant decrease in the content of DAA in the muscles of Qihe crucian carp, indicating that the flavor of the muscles might be affected in an alkaline water environment [23]. Although the TAA and NEAA decreased progressively under alkalinity stress, their ratios remained within the ideal model recommended by WHO/FAO for protein nutrition (EAA/TAA = 40.00%, EAA/NEAA > 60.00%), indicating that Qihe crucian carp under alkalinity stress remains a high-quality protein source [54].

As fundamental lipid components, fatty acids provide metabolic energy and play critical biological roles [55]. Although alkalinity stress significantly reduced total PUFA, n-3 PUFA, and n-6 PUFA contents in the muscle of Qihe crucian carp, the n-3/n-6 ratios in all experimental groups (0.31, 0.38, 0.36, respectively) exceeded the FAO/WHO recommended minimum of 0.2 for health benefits [56]. This indicated that the muscle nutritional value was largely preserved under medium-high alkalinity culture conditions.

The water-holding capacity of muscle is a critical determinant of both product quality and processability [57]. Generally, a higher water-holding capacity is positively correlated with slower protein degradation, better flavor retention, and an extended storage period [58]. Compared to the control group, the CA40 group demonstrated an increase in centrifugal loss and drip loss, along with a decrease in pH value. This indicated that alkalinity stress compromised the water-holding capacity of Qihe crucian carp muscle, consequently diminishing its quality. Previous work on largemouth bass demonstrated that the increased muscle fiber density enhanced water retention by tightening the cellular matrix [33]. Our results revealed that both water-holding capacity and muscle fiber density of Qihe crucian carp significantly decreased under alkalinity stress. This result was similar to the findings in largemouth bass, where muscle fiber density was positively correlated with water-holding capacity [33]. Therefore, we speculated that the alkalinity stress reduced the muscle fiber density of Qihe crucian carp, leading to a looser grid structure between muscle cells. This structural change likely impaired the effective retention of water molecules within the muscle tissue.

For fish muscle, physical characteristics are typically represented by sensory indicators such as the hardness, chewiness, and springiness, while the structural characteristics are mainly indicated by the structure of muscle fibers [59]. A previous study on largemouth bass reported that alkalinity stress significantly enhanced the textural properties, including the chewiness, cohesiveness, resilience, hardness and gumminess [23]. Structurally, alkalinity stress was found to significantly reduce muscle fiber diameter and cross-sectional area, while increasing muscle fiber density in largemouth bass [23]. This indicated that the structural characteristics of muscle may influence its physical properties. Nevertheless, to date, the understanding of how alkalinity affects the textural properties of fish muscle remains limited. Another study on largemouth bass also found that muscle fiber density was positively correlated with hardness [60]. The results of this study indicated that alkalinity stress significantly reduced the muscle hardness of Qihe crucian carp, which may be primarily due to a decrease in muscle fiber density. Muscle fiber density is a key determinant of meat quality, with higher density generally correlating with superior texture in a given species [61]. Structurally, alkalinity stress led to a reduction in both the long diameter and density of muscle fibers in Qihe crucian carp. In summary, alkalinity stress induced modifications in the physical and structural characteristics of Qihe crucian carp muscle, thereby compromising its quality.

While our study confirmed the effects of alkalinity stress on the growth performance and muscle quality of Qihe crucian carp, it was important to acknowledge that the complex ionic composition of natural saline-alkaline water was not fully replicated in our experimental system. In the future, comprehensive physiological indicators after alkalinity stress will be thoroughly examined, including blood biochemistry and physiological analyses, as well as gene expression profiling analysis. Omics approaches, such as transcriptomics and metabolomics, should also be employed to further elucidate the molecular mechanisms through which alkalinity stress affects the growth and nutritional quality of fish. In addition, genomics-assisted breeding (GAB) should be emphasized in future research, as it represents an effective strategy to genetically enhance alkalinity tolerance, growth performance, and muscle quality in farmed fish. Furthermore, outdoor aquaculture experiments are needed to be conducted to systematically evaluate the combined effects of alkalinity stress with other environmental factors (e.g., salinity and temperature), thereby providing a more reliable data foundation for saline-alkaline water aquaculture of Qihe crucian carp. Moreover, it is necessary to develop economically effective feed additives to alleviate the adverse effects of alkalinity stress on the growth performance and muscle quality of Qihe crucian carp.

## 5. Conclusions

The study provided a multifaceted analysis of how medium-high carbonate alkalinity (20–40 mmol/L) influenced Qihe crucian carp, encompassing assessments of growth performance, morphology traits, physiology responses, histology structures, and muscle nutritional quality. The results indicated that Qihe crucian carp had a high alkalinity tolerance. The alkalinity ≤ 40 mmol/L did not affect the SR. However, alkalinity stress significantly impaired both growth performance and feed efficiency. Qihe crucian carp may adapt to the alkalinity stress through adjustments in serum ion concentrations (Na^+^ and Ca^2+^), gill enzyme activities (NKA and CMA), and gill histology. Alterations in muscle nutritional components (amino acids, fatty acids), physical properties, and microstructure induced by alkalinity stress ultimately led to a reduction in overall muscle nutritional quality. However, the muscles of Qihe crucian carp cultured in the medium-high alkalinity waters still belong to the high-quality protein recommended by WHO/FAO. Considering both growth performance and muscle quality, the suitable alkalinity for the practical saline-alkaline water aquaculture of Qihe crucian carp should be controlled below 20 mmol/L.

## Figures and Tables

**Table 1:** Comparison of standardized external morphological characteristics in Qihe crucian carp *Carassius carassius* after 60 d alkalinity stress.

Items (%)	Control	CA20	CA40
FL/BL	121.21 ± 1.08 ^b^	121.19 ± 0.52 ^b^	122.85 ± 1.61 ^a^
HL/BL	27.04 ± 1.07	26.56 ± 0.99	26.84 ± 1.00
TL/BL	50.40 ± 1.77	48.16 ± 2.06	50.16 ± 2.20
BH/BL	34.62 ± 0.38 ^a^	33.68 ± 0.85 ^a^	32.27 ± 1.04 ^b^
HLBE/BL	20.54 ± 0.69 ^b^	21.62 ± 0.99 ^a^	21.94 ± 0.77 ^a^
BT/BL	18.95 ± 0.61 ^a^	18.30 ± 0.79 ^ab^	17.64 ± 0.64 ^b^
SL/BL	7.70 ± 0.50 ^a^	5.54 ± 0.24 ^b^	5.72 ± 0.41 ^b^
DBE/BL	15.56 ± 0.60 ^a^	15.22 ± 0.47 ^a^	14.63 ± 0.75 ^b^
CL/BL	24.21 ± 1.54	24.39 ± 1.64	23.71 ± 1.50
CFL/BL	20.46 ± 0.66	20.23 ± 0.47	21.37 ± 0.98
CPL/BL	16.22 ± 1.44	15.97 ± 1.19	16.65 ± 1.73
CPH/BL	14.68 ± 0.40 ^a^	14.22 ± 0.44 ^b^	14.05 ± 0.31 ^b^
FL/BL	121.21 ± 1.08 ^b^	121.19 ± 0.52 ^b^	122.85 ± 1.61 ^a^

All external morphological traits were standardized by dividing them by body length. BL: body length; FL: full length; HL: head length; TL: trunk length; BH: body height; HLBE: head length behind eyes; BT: body thickness; SL: snout length; DBE: distance between eyes; CL: caudal length; CFL: caudal fin length; CPL: caudal peduncle length; CPH: caudal peduncle height. Data are presented as mean ± SD, different superscript letters within a same line denote significant differences (*p* < 0.05).

**Table 2:** Proximate composition (% wet weight) of the muscle in Qihe crucian carp *Carassius carassius* after 60 d alkalinity stress.

Items	Control	CA20	CA40
Moisture	77.86 ± 0.02 ^a^	77.52 ± 0.14 ^b^	78.07 ± 0.08 ^a^
Crude ash	1.12 ± 0.05 ^b^	1.19 ± 0.90 ^b^	1.58 ± 0.05 ^a^
Crude fat	3.58 ± 0.16 ^a^	3.38 ± 0.11 ^ab^	3.16 ± 0.05 ^b^
Crude protein	20.30 ± 0.16 ^a^	19.88 ± 0.28 ^ab^	19.44 ± 0.15 ^b^

Data are presented as mean ± SD, different superscript letters within a same line denote significant differences (*p* < 0.05).

**Table 3:** Muscle amino acid contents (g/100 g dry weight) in Qihe crucian carp *Carassius carassius* after 60 d alkalinity stress.

Amino Acid	Control	CA20	CA40
Aspartic acid ^☼^	2.02 ± 0.02 ^a^	1.97 ± 0.01 ^b^	1.89 ± 0.02 ^c^
Glutamic acid ^☼^	3.06 ± 0.02 ^a^	2.99 ± 0.02 ^b^	2.90 ± 0.01 ^c^
Serine	0.71 ± 0.02 ^a^	0.71 ± 0.01 ^a^	0.68 ± 0.01 ^b^
Argnine ^●^	1.29 ± 0.05 ^a^	1.22 ± 0.01 ^b^	1.21 ± 0.01 ^b^
Glycine ^☼^	0.98 ± 0.01 ^a^	0.98 ± 0.02 ^a^	0.92 ± 0.02 ^b^
Threonine ^●^	0.93 ± 0.01 ^a^	0.93 ± 0.01 ^a^	0.89 ± 0.01 ^b^
Proline	0.69 ± 0.01 ^a^	0.69 ± 0.01 ^a^	0.66 ± 0.02 ^b^
Alanine ^☼^	1.25 ± 0.02 ^a^	1.24 ± 0.01 ^a^	1.20 ± 0.01 ^b^
Valine ^●^	0.90 ± 0.01	0.89 ± 0.01	0.89 ± 0.03
Methionine ^●^	0.52 ± 0.01 ^a^	0.49 ± 0.01 ^b^	0.49 ± 0.01 ^b^
Cysteine	0.18 ± 0.00	0.16 ± 0.00	0.15 ± 0.00
Isoleucine ^●^	0.86 ± 0.03	0.84 ± 0.03	0.81 ± 0.03
Leucine ^●^	1.53 ± 0.02 ^a^	1.49 ± 0.02 ^b^	1.45 ± 0.01 ^c^
Phenylalanine ^●^	0.95 ± 0.01 ^a^	0.93 ± 0.01 ^b^	0.88 ± 0.01 ^c^
Histidine ^●^	0.59 ± 0.02 ^a^	0.61 ± 0.00 ^a^	0.51 ± 0.02 ^b^
Lysine ^●^	2.03 ± 0.01 ^a^	1.99 ± 0.02 ^a^	1.94 ± 0.04 ^b^
Tyrosine ^●^	0.65 ± 0.00 ^a^	0.63 ± 0.01 ^b^	0.60 ± 0.01 ^c^
TAA	19.12 ± 0.19 ^a^	18.75 ± 0.12 ^b^	18.06 ± 0.17 ^c^
EAA	7.72 ± 0.07 ^a^	7.55 ± 0.07 ^b^	7.35 ± 0.06 ^c^
NEAA	11.40 ± 0.14 ^a^	11.19 ± 0.06 ^a^	10.71 ± 0.12 ^b^
DAA	7.30 ± 0.06 ^a^	7.18 ± 0.04 ^b^	6.90 ± 0.06 ^c^
EAA/TAA	40.40 ± 0.19 ^ab^	40.29 ± 0.09 ^b^	40.69 ± 0.16 ^a^
EAA/NEAA	67.77 ± 0.53 ^ab^	67.48 ± 0.25 ^b^	68.60 ± 0.45 ^a^

^●^: essential amino acids; ^☼^: delicious amino acids; TAA: total amino acids; EAA: essential amino acids; NEAA: non-essential amino acids; DAA: delicious amino acids. The data are presented as means ± SD, and the data in the same row with different superscript letters are significantly different (*p* < 0.05).

**Table 4:** Water-holding capacity and pH of Qihe crucian carp *Carassius carassius* muscle after 60 d alkalinity stress.

Items	Control	CA20	CA40
Drip loss (%)	10.39 ± 1.29 ^b^	11.07 ± 0.88 ^ab^	12.61 ± 0.45 ^a^
Centrifugal loss (%)	5.74 ± 0.18 ^b^	5.97 ± 0.84 ^b^	7.46 ± 0.22 ^a^
Liquid loss (%)	19.47 ± 2.34	19.38 ± 0.26	21.89 ± 0.75
Stored loss (%)	4.25 ± 0.21 ^a^	3.41 ± 0.31 ^b^	3.75 ± 0.19 ^b^
Cooked rate (%)	64.01 ± 2.82 ^b^	69.48 ± 1.09 ^a^	74.24 ± 2.94 ^a^
Frozen leakage (%)	1.56 ± 0.02 ^b^	2.55 ± 0.37 ^a^	2.38 ± 0.14 ^a^
pH value	6.27 ± 0.21 ^a^	5.96 ± 0.05 ^b^	5.89 ± 0.07 ^b^

Data are presented as mean ± SD, and the data in the same row with different superscript letters are significantly different (*p* < 0.05).

## Data Availability

The data presented in this study are available on request from the corresponding author.

## Abbreviations

The following abbreviations are used in this manuscript:

EPCs	epithelial cells
PCs	pillar cells
MRCs	mitochondria-rich cells
NKA	Na^+^/K^+^-ATPase
CMA	Ca^2+^/Mg^2+^-ATPase
TAA	total amino acid
EAA	essential amino acid
DAA	delicious amino acid
FI	Feed intake
FBW	final body weight
SR	survival rate
SGR	specific growth rate
FR	feeding rate
WGR	weight gain rate
ROS	reactive oxygen species
FCR	feed conversion ratio
BW	body weight
FL	full length
HL	head length
HLBE	head length behind eyes
DBE	distance between eyes
BT	body thickness
BL	body length
SL	snout length
BH	body height
CL	caudal length
CPL	caudal peduncle length
CFL	caudal fin length
CPH	caudal peduncle height
TL	trunk length
CF	condition factor
VSI	viscerosomatic index
HSI	hepatosomatic index
MC	meat content
PFA	paraformaldehyde
PBS	phosphate-buffered saline
H&E	hematoxylin and eosin
SFA	saturated fatty acid
MUFA	monounsaturated fatty acids
PUFA	polyunsaturated fatty acids
EPA	eicosapentaenoic acid
DHA	docosahexaenoic acid
MCs	mucous cells
PVCs	pavement cells
BCs	blood cells
GAB	genomics-assisted breeding
